# Somatic genome alterations in relation to age in lung squamous cell carcinoma

**DOI:** 10.18632/oncotarget.25848

**Published:** 2018-08-14

**Authors:** Stefano Meucci, Ulrich Keilholz, Daniel Heim, Frederick Klauschen, Stefano Cacciatore

**Affiliations:** ^1^ Charité Comprehensive Cancer Center, Charité University Hospital, Berlin, Germany; ^2^ Institut für Pathologie, Charité University Hospital, Berlin, Germany; ^3^ Imperial College Parturition Research Group, Division of the Institute of Reproductive and Developmental Biology, Imperial College London, London, England, UK; ^4^ International Centre for Genetic Engineering and Biotechnology, Cancer Genomics Group, Cape Town, South Africa

**Keywords:** lung squamous cell carcinoma, aging, somatic mutations, copy number variations, methylation

## Abstract

Lung squamous cell carcinoma (LUSC) is the most common cause of global cancer-related mortality and the major risk factors is smoking consumption. By analyzing ∼500 LUSC samples from The Cancer Genome Atlas, we detected a higher mutational burden as well as a higher level of methylation changes in younger patients. The SNPs mutational profiling showed enrichments of smoking-related signature 4 and defective DNA mismatch repair (MMR)-related signature 6 in younger patients, while the defective DNA MMR signature 26 was enriched among older patients. Furthermore, gene set enrichment analysis was performed in order to explore functional effect of somatic alterations in relation to patient age. Extracellular Matrix-Receptor Interaction, Nucleotide Excision Repair and Axon Guidance seem crucial disrupted pathways in younger patients. We hypothesize that a higher sensitivity to smoking-related damages and the enrichment of defective DNA MMR related mutations may contribute to the higher mutational burden of younger patients. The two distinct age-related defective DNA MMR signatures 6 and 26 might be crucial mutational patterns in LUSC tumorigenesis which may develop distinct phenotypes. Our study provides indications of age-dependent differences in mutational backgrounds (SNPs and CNVs) as well as epigenetic patterns that might be relevant for age adjusted treatment approaches.

## INTRODUCTION

Lung cancer is the most common cause of global cancer-related mortality and the major risk factors are smoking consumption and occupational exposure to carcinogens [1]. The two major histological classes are non-small-cell lung cancer (NSCLC) and small-cell lung cancer (SCLC). NSCLCs mostly comprise lung adenocarcinomas (LUAD) and lung squamous carcinomas (LUSC) [2], characterized by largely distinct mutational patterns [3].

The mutational landscape present in a cancer genome is the cumulative result of endogenous and/or exogenous mutational processes (e.g., smoking), constant or sporadic and with different strengths along patient ageing [4–7]. Therefore, multiple mutational processes are operative resulting in jumbled composite signatures and tumor characteristics vary between patients of different ages [7–9]. From the Catalogue Of Somatic Mutations In Cancer (COSMIC) which includes 10,952 exomes and 1,048 whole-genomes across 40 distinct types of human cancer [10], 30 different mutational signatures were identified and publicly released (http://cancer.sanger.ac.uk/cosmic/signatures). Each signature is characterized by the contribution of different factor (e.g., smoking, age, sex). Signature 1 (SI1) characterized by C>T transitions at CpG sites due to the deamination of 5-methylcytosine was associated to mutational processes related to the ageing [4–6, 11]. While Signature 4 (SI4) associated with C>A transversions was found in cancers in which tobacco smoking increases risk and mainly in those derived from cells directly exposed to the tobacco smoke. According to the SI4 pattern, LUSC patients can be classified by the “transversion status” in order to study high and low mutational rate profiles [3]. Past studies hypothesized that chemicals of tobacco smoke increases the speed with which these mutations accumulate [12]. Although the age at diagnosis of lung tumors is very closely correlated with the duration of smoking [13, 14], a previous study performed on 34 tumor types of the TCGA dataset [15], showed significant negative correlations between SNPs and patient age only in LUSC and LUAD. While 29 tumor types exhibited positive correlations, among which the smoking-related tumors such as HNSCC [15, 16]. Therefore the hypothesis of the “mutator phenotype”, which is a tumor harboring mutations in DNA polymerases and DNA repair genes [15, 17], has to be taken into account.

Furthermore, Copy Number Variations (CNVs) play also important roles in the development of cancer showing an association with ageing in terms of longevity, healthy aging, and aging-related pathologies [18–20]. Although the number of studies about CNVs and ageing are very limited, age-related CNVs increase observed in human blood cell genomes [21, 22] suggests that CNVs could play a key role even in LUSC.

Moreover, epigenomic alteration is now increasingly recognized as part of aging and its associated pathologic phenotypes as cancer [23]. There is ample evidence for changes in DNA methylation patterns at CpG sites during development and aging, driving essential somatic functions. A general demethylation is linked with aging which may reflects some deficiency in maintenance re-methylation. The epimutation rate appears to be almost 100,000 times the mutation rate and aberrant DNA methylation can predispose to malignancy [22, 24, 25].

This study aims to provide better insight into the underlying genetic and epigenetic patterns of LUSC in relation to patient age. To this end, we investigated the relationships between patient age and the average number of SNPs, CNVs and methylation changes as well as the SNPs profiling and the respective correlation to the previously defined signatures in COSMIC. Furthermore, we performed gene-specific correlation analysis in relation to patient age with a particular focus on the significantly mutated genes in LUSC [3] and the most frequently mutated DNA repair genes in lung cancer [26]. Finally, gene set enrichment analysis was performed in order to explore functional effect of somatic alterations in relation to patient age.

The current study may pave the way for future studies of molecular tumorigenesis in relation to human ageing and underlines the need to consider age-adjusted treatments not only based on age and morbidity of older patients, but also on differences in tumor biology.

## RESULTS

### Somatic alterations and patient age

Genome-wide mutations and epigenomic changes are expected to varying among tumor subtypes showing a different distribution across age. To characterize these distinct distribution patterns, we firstly estimated the global number of SNPs, CNVs, and methylation changes at CpG sites for 504 samples across LUSC cancer cohort available through The Cancer Genome Atlas (TCGA). We used the Spearman’s rank correlation coefficient to explore the relation between the number of SNPs, CNVs and methylation changes with patient age.

**Figure 1:**
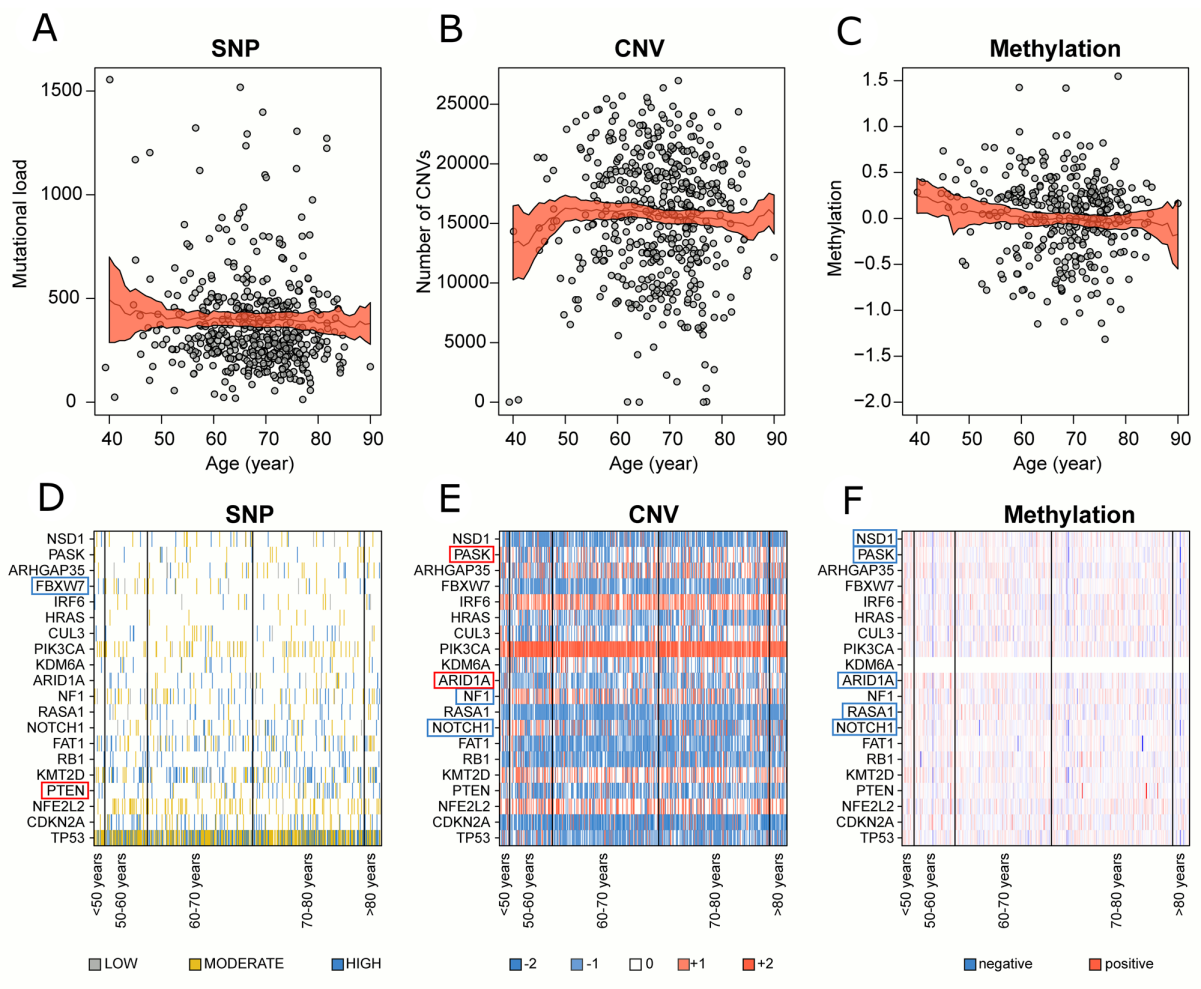
Correlation between genomic alterations and patient age in global cohort Number of **(A)** SNPs, **(B)** CNVs and **(C)** methylation changes with their relative 95% confidence interval for each patient distributed along patient age. Medians (black line) and their relative 95% confidence interval (red area) were calculated locally in a range of ±10 years. **(D)** SNPs, **(E)** CNVs and **(F)** methylation changes profile of the 20 significantly mutated genes in LUSC. Significantly positive and negative correlated genes were highlighted in red and blue respectively.

The global SNPs load showed a slightly negative correlation with patient age (Table 1), which indicated a higher mutational rate among younger patients (Figure 1A). Then, we classified SNPs according to their expected biological effect as low, moderate, or severe (as shown in Supplementary Table 1) and we identified the genes with at least a severe or moderate mutation. We reported a lower correlation between the age and the number of genes with disruptive mutations (*rho*=-0.08, *p*=0.077, *FDR*=0.26). The global CNVs load showed no correlation with patient age (Figure 1B). While methylation changes were negatively correlated with patient age (*rho*=-0.11, *p*=0.030, *FDR*=0.23) displaying a higher level of methylation at CpG sites among younger patients (Figure 1C).

**Table 1:** SNPs loads correlations with patient age

Classification	Patients n.	rho [95%CI]	p-value	FDR
*Global*	480	-0.09 [-0.19 0]	4.53×10^-2^	1.81×10^-1^
*Transversion Status*				
High	387	-0.11 [-0.22 -0.01]	2.60×10^-2^	1.56×10^-1^
Low	84	0.15 [-0.05 0.34]	1.87×10^-1^	3.21×10^-1^
*Tobacco smoking history indicator*				
Lifelong non-smokers	18	0.11 [-0.41 0.61]	6.54×10^-1^	7.85×10^-1^
Current smokers	131	-0.12 [-0.29 0.05]	1.66×10^-1^	3.21×10^-1^
Current reformed smokers for >15 yrs	78	-0.19 [-0.38 0.03]	9.88×10^-2^	2.96×10^-1^
Current reformed smokers for < or = 15 yrs	236	-0.09 [-0.22 0.05]	1.59×10^-1^	3.21×10^-1^
Current reformed smokers, duration not specified	5	-0.1 [-1 1]	9.50×10^-1^	9.50×10^-1^
*Ajcc pathologic tumor stage*				
1	233	-0.07 [-0.19 0.06]	3.13×10^-1^	4.70×10^-1^
2	153	0.02 [-0.13 0.19]	7.66×10^-1^	8.36×10^-1^
3	83	-0.35 [-0.53 -0.15]	1.12×10^-3^	1.34×10^-2^
4	7	-0.29 [-0.96 0.62]	5.56×10^-1^	7.41×10^-1^

Correlations between the SNPs loads and patient age for each patient sub-group established according to the patient characteristic evaluated in our study, such as tobacco exposure data (i.e., tobacco smoking history indicator), tumor staging (i.e., ajcc pathologic tumor stage), and mutational rate profile (i.e., transversion status).

We repeated the analysis on patient sub-cohorts established according to the tobacco exposure data (i.e., tobacco smoking history indicator), tumor staging (i.e., ajcc pathologic tumor stage), and mutational rate profile (i.e., transversion status) in order to explore the influence of patient features on the relation among SNPs, CNVs, and methylation changes with patient age. The analysis of sub-cohort with a high mutational load (i.e., transversion-high status) showed a negative correlation between the SNPs load and patient age while no correlations were detected in the low mutational load sub-cohort (i.e., transversion low status) (Table 1). The results regarding CNVs and methylation changes were fully reported in Supplementary Table 2.

### Gene-specific alterations enrichment along patient ageing

The Spearman’s rank correlation was computed between SNPs, CNVs, and methylation changes in each gene and patient age, we reported the results in Supplementary Table 3. A special focus was placed on the 20 significantly mutated genes previously found in LUSC [3] (Supplementary Table 4, Figure 1D–1F). A negative correlation between patient age and both CNVs (*rho*=-0.13, *p*=0.005, *FDR*=0.16) and methylation changes (*rho*=-0.14, *p*=0.006, *FDR*=0.06) was detected on NOTCH1, while no SNPs correlation was displayed. A significantly higher level of methylation at CpG sites in younger patients was as well exhibited in RASA1 (*rho*=-0.19, *p*=0.0002, *FDR*=0.01), ARID1A1 (*rho*=-0.22, *p*=0.00005, *FDR*=0.006), PASK (*rho*=-0.11, *p*=0.04, *FDR*=0.16) and NSD1 (*rho*=-0.13, *p*=0.02, *FDR*=0.09).

In order to explore the hypothesis of possible mutator phenotypes contributing to the high mutational rate detected among younger patients, we analyzed whether mutations harboring on the top 20 frequently mutated DNA repair genes in lung cancer [26] might have a significant impact on the SNPs load. For each of them, the Wilcoxon test was performed to compare the mutational load of the patient sub-cohorts exhibiting the somatic alterations against the wild-type patient groups (Supplementary Table 5). The percentage of patients which have at least one of the genes mutated was >83% in each age-group. The mutator phenotype had a significant impact on the mutational load in 60-70 and 70-80 age classes. Therefore the analysis was repeated grouping the patient global cohort in younger and older than 60 years old. While only 3 genes were significant in ≤60 years old patients, 14 out of 20 genes had a significant impact on the mutational load in >60 years old patients.

### Age-related COSMIC signatures

Somatic mutation profile is the sum of multiple mutation processes, such as the intrinsic infidelity of the DNA replication machinery, exogenous or endogenous mutagen exposures, enzymatic modification of DNA, and defective DNA repair. In order to analyze each mutation process separately, we correlated the patient age with single nucleotide variants (Supplementary Table 6) and COSMIC signatures (Supplementary Table 7) using the Spearman’s rank correlation. Additionally, the Wilcoxon Rank-Sum test was performed to evaluate the differences between each age group (i.e., <50, 50-60, 60-70, 70-80, >80) and the rest of the cohort.

**Figure 2:**
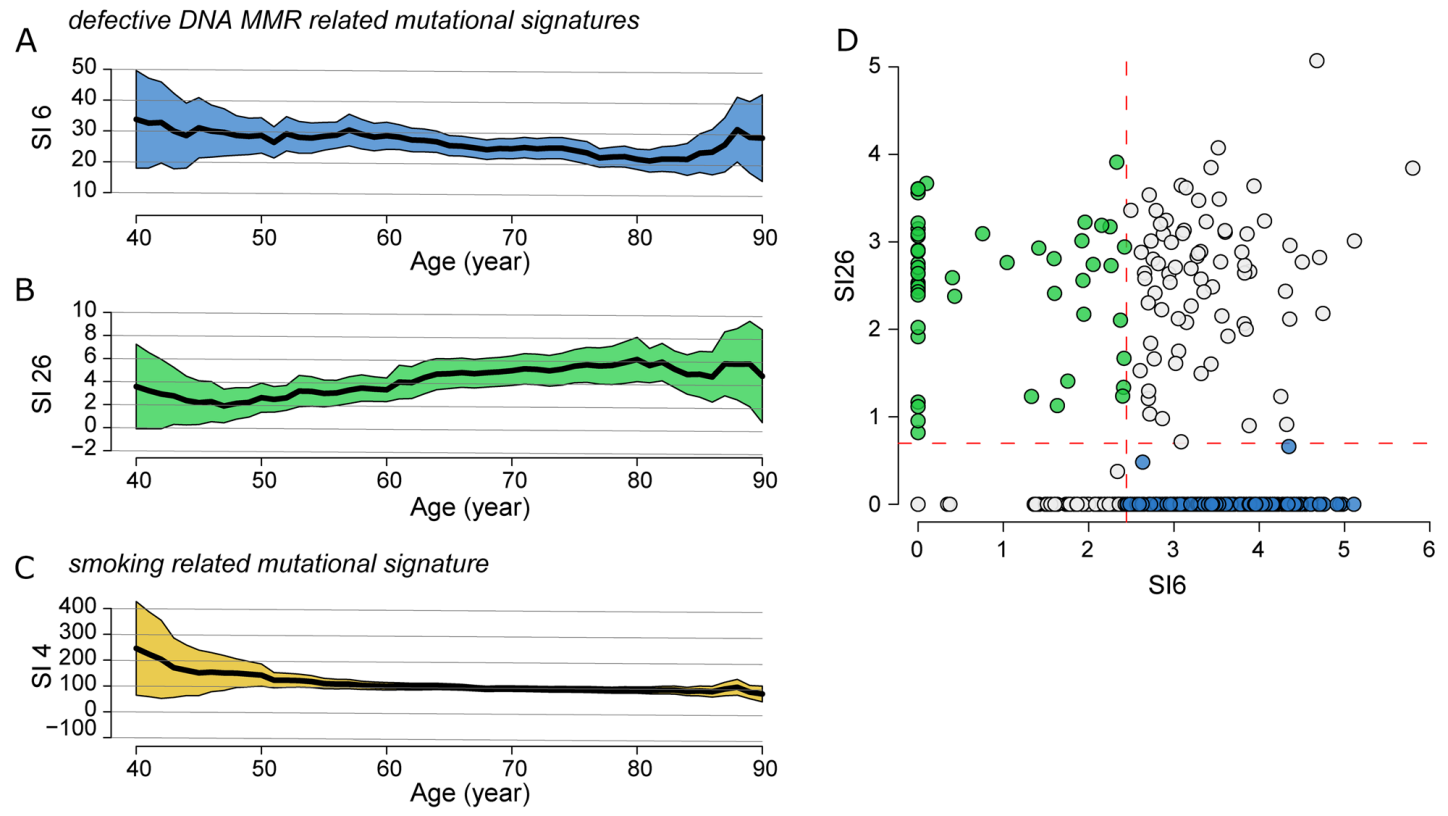
Correlation of SNPs profiling and patient age in global cohort Correlation between defective DNA MMR **(A)** SI6 and **(B)** SI26, and smoking related **(C)** SI4 with patient age. Medians (black line) and their relative 95% confidence interval (colored area) were calculated locally in a range of ±10 years. **(D)** Classification of the overall LUSC cohort into four subgroups using the mean values (dashed red lines) of SI6 and SI26 as threshold: high-SI6/high-SI26, low-SI6/high-SI26 (green circle), high-SI6/low-SI26 (blue circle) and low-SI6/low-SI26. The values are converted as log(x+1).

The defective DNA mismatch repair (MMR)-related signature 6 (SI6) was negatively correlated (*rho*=-0.13, *p*=0.004, *FDR*=0.12) with the patient age (Figure 2A) while the signature 26 (SI26) as well associated with defective DNA MMR, was positively correlated (*rho*=0.11, *p*=0.013, *FDR*=0.20) with the patient age (Figure 2B). Both signatures showed similar trend in the transversion-high sub-cohort. The smoking-related SI4 was negatively correlated (*rho*=-0.11, *p*=0.02, *FDR*=0.21) with patient age (Figure 2C), showing higher values in the ≤50 and 51-60 age groups (Supplementary Table 7). No correlation was detected for the age-related SI1.

In order to study the patient sub-cohorts, which predominantly exhibit SI26 and SI6, we divided the overall LUSC cohort into four subgroups using the mean values of SI6 and SI26 as threshold (Figure 2D): high-SI6/high-SI26 (77/480=16.0%), low-SI6/high-SI26 (55/480=11.0%), high-SI6/low-SI26 (223/480=45.8%), and low-SI6/low-SI26 (130/480=27.1%). We selected and characterized the low-SI6/high-SI26 and high-SI6/low-SI26 subgroups (Supplementary Table 8). The patients age of the low-SI6/high-SI26 cohort was significantly higher than the high-SI6/low-SI26 cohort (Wilcoxon Rank-Sum test: *p*=0.005).

### Gene set enrichment analysis

On the basis of the previous analysis, the LUSC mutation profile in relation to ageing is characterized by two major defective DNA MMR-related signatures (i.e., SI6 and SI26). To study the molecular effects of these signatures independently, we projected the SNPs, CNVs and DNA methylation values from the high-SI6/low-SI26 and low-SI6/high-SI26 subtypes into the space of the 186 Kyoto Encyclopedia of Genes and Genomes (KEGG) pathways by means of single-sample gene set enrichment analysis (ssGSEA) (Supplementary Table 9) [27].

**Figure 3:**
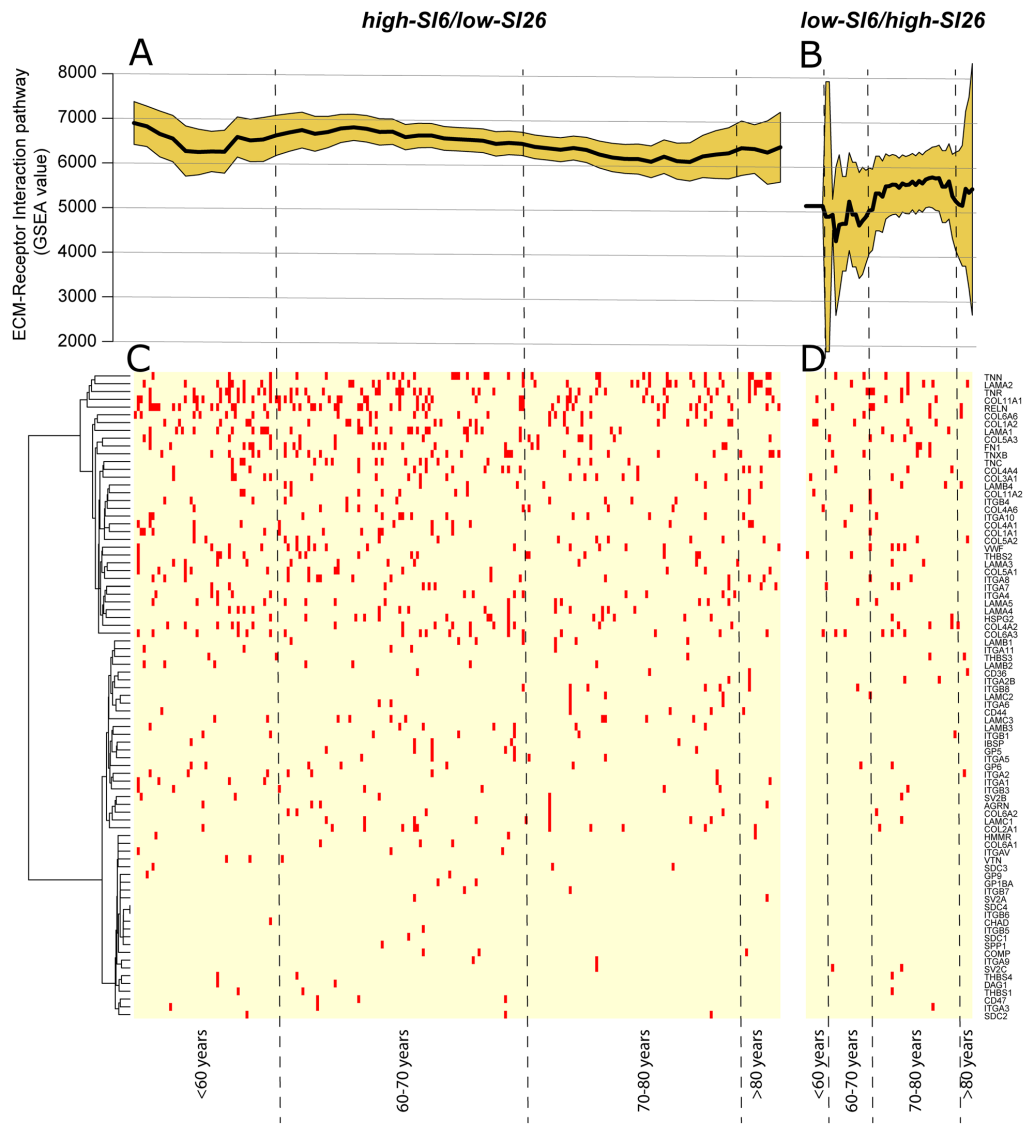
**(A)** GSEA value of “ECM-Receptor Interaction” pathway in high-SI6/low-SI26 and **(B)** low-SI6/high-SI26 patient sub-cohorts. Unsupervised hierarchical clustering of SNPs frequencies of genes involved in the “ECM Receptor Interaction” pathway (according to the KEGG database) in **(C)** high-SI6/low-SI26 and **(D)** low-SI6/high-SI26.

Using the Wilcoxon Rank-Sum test, we reported as major significant differences, that Extracellular Matrix (ECM)-Receptor Interaction pathway (*p*=0.0002, *FDR*=0.04) was significantly enriched of SNPs while the Nucleotide Excision Repair pathway was enriched in CNVs (*p*=0.0007, *FDR*=0.14) in high-SI6/low-SI26 sub-cohort (Figure 3). The Regulation of Autophagy pathway (*p*=0.0006, *FDR*=0.06) showed an enrichment of SNPs in low-SI6/high-SI26 patient sub-cohort. Using the Spearman’s Rank Correlation Coefficient, we detected a negative correlation between SNPs harboring on ECM Receptor Interaction pathway and patient age (*rho*=-0.16, *p*=0.016, *FDR*=0.73) in high-SI6/low-SI26 sub-cohort. In Figure 3, the GSEA values of “ECM-Receptor Interaction” pathway were reported for both (Figure 3A) high-SI6/low-SI26 and (Figure 3B) low-SI6/high-SI26 patient sub-cohorts in order to visualize the different trends. Unsupervised hierarchical clustering of SNPs frequencies of genes involved in the “ECM Receptor Interaction” pathway (according to the KEGG database) was added in order to report the pathway mutation profile (Figure 3C–3D).

When evaluating the global cohort, we detected a significant negative correlation between patient age and SNPs harboring on “Axon-Guidance” (*rho*=-0.15, *p*=0.0007, *FDR*=0.14) and ECM Receptor Interaction (*rho*=-0.13, *p*=0.003, *FDR*=0.16) pathways, particularly in the 51-60 age group. Furthermore, the Axon-Guidance (*rho*=-0.16, *p*=0.001, *FDR*=0.12) pathway was the only negatively enriched pathway in transversion-high sub-cohort (Supplementary Table 10).

## DISCUSSION

We identified a slightly higher SNPs load among younger patients of the TCGA LUSC patient cohort confirming a previous study [15]. In particular, the correlation was higher in tumors with high mutational burden. Since the correlation was not robust, we believe that our results must be evaluated in an independent cohort to confirm higher mutational rate in younger patients. Interestingly, a higher overall methylation rate at CpG sites was as well detected among younger patients. Although the knowledge is still limited, numerous studies showed that CpG methylation plays an important role in maintaining gene silencing. Several studies have revealed that tumor suppressor gene promoter hypermethylation is noted in tumor cells [28]. However, normal non-proliferative cells also showed gene promoter hypermethylation as age increases [29, 30]. Age-dependent hypermethylation at CpGs was observed to be enriched with DNA binding factors and transcription factors, therefore the dysregulation can simultaneously affect several biological processes [31, 32]. On the contrary Heyn *et al.* [32] revealed that centenarians exhibit lower DNA methylation levels compared with newborns. Therefore, the higher methylation level at CpG sites among younger patients detected in our study might comprise both aberrations and normal age-related patterns. We detected 5 out of 20 significantly mutated genes in LUSC (NOTCH1, RASA1, ARID1A1, PASK, NSD1) exhibiting a significantly higher methylation levels in younger patients. CNVs enrichment was as well detected in NOTCH1 among younger patients. NOTCH1 is one of the highly significant mutated genes in Cancer. Cross-talking with many other critical cancer genes and pathways, NOTCH1 is involved in multifaceted regulation of cell survival, proliferation, tumor angiogenesis, and metastasis. A recent study observed that with long-term smoking exposure, the DNA sequence suffers persistent miscoding that triggers epigenetic changes in NOTCH1 [33]. Therefore NOTCH1 aberrations might be involved in the peculiar higher mutational burden of younger LUSC patients.

Mutator phenotypes might develop in LUSC tumorigenesis [15], therefore we evaluated the mutational profile of the top 20 frequently mutated DNA repair genes in lung cancer [26]. No significant differences in mutation frequencies were detected among the age classes. More than 83 % of the patients harbored at least one of the genes mutated in all age classes. Thus, mutator phenotypes seem evenly distributed along patient ageing, contributing to the overall high mutational burden in LUSC patients. On the contrary, the impact of these mutations on the mutational load was significantly higher in >60 years old patients. Therefore, mutator phenotypes might have different consequences in relation to ageing processes.

The overall SNPs mutational profiling and the corresponding correlations with COSMIC signatures showed an enrichment of the smoking-related signature (i.e., SI4) among younger patients. Past studies described a similar scenario showing that despite maintained carcinogen exposure, tumors from smokers showed a relative decrease in smoking-related mutations over time [34, 35]. Therefore, younger patients may develop higher sensitivity to smoking-related mutations. The defective DNA MMR SI6 and SI26 were as well significantly correlated with patient age. The SI6, characterized predominantly by C>T at NpCpG sites (any nucleotide followed by C followed by G), was enriched in younger patients. While the SI26, mostly composed of T>C transitions, was enriched in older patients. Both SI6 and SI26 are found in microsatellite unstable tumors with high numbers of small (shorter than 3bp) insertions and deletions at mono/polynucleotide repeats [36, 37]. The role of MMR system is to recognize and repair erroneous insertion, deletion, and mis-incorporation of bases arising during DNA replication and homologous recombination, as well as repairing some forms of DNA damage. Given the importance of these processes in the maintenance of genomic stability, DNA MMR deficiency might leads to hypermutation [38, 39]. A recent study showed that out of a large number of DNA repair deficiencies analyzed, MMR deficiency leads to the by far highest mutation rate [36]. Our results suggest that different causing factors might contribute to MMR system aberrations along patient ageing. Therefore we performed gene set enrichment analysis in patient sub-cohorts which predominantly exhibit SI6 or SI26. We identified the SNPs enrichment in ECM-Receptor Interaction pathway among younger patients of high-SI6/low-SI26 sub-cohort. The ECM-Receptor Interaction pathway is structurally and functionally involved in interactions at the ECM which lead to a direct or indirect control of cellular activities such as cell migration, differentiation, proliferation, and apoptosis [40–42]. Aberrant ECM may promote genetic instability and might compromise DNA repair pathways necessary to prevent malignant transformation [40]. Furthermore, we identified an enrichment of CNVs in Nucleotide Excision Repair (NER) pathway in high-SI6/low-SI26 sub-cohort. Since the NER system is primarily responsible for detecting and removing bulky DNA lesions induced by tobacco smoke in the respiratory tract [43], SNPs in NER protein-encoding genes may contribute to the higher sensitivity to smoking consumption detected in younger patients. Early studies identified associations with lung cancer risk in selected mutated NER genes (ERCC1-6, LIG1, POLE, XPA, and XPC genes) [44–47].

The low-SI6/high-SI26 sub-cohort was enriched in SNPs disruptions of Regulation of Autophagy pathway involved in lysosome-dependent degradation processes. On one hand, autophagy has been shown to regulate some of the DNA repair proteins after DNA damage by maintaining the balance between their synthesis, stabilization, and degradation. One the other hand, some evidence has demonstrated that some DNA repair molecules have a crucial role in the initiation of autophagy [48, 49]. Therefore, disruption of Regulation of Autophagy pathway might contribute to the defective DNA MMR system in low-SI6/high-SI26 patient sub-cohort.

Considering the “global” cohort, SNPs harboring on genes involved in ECM-Receptor Interaction and Axon Guidance pathways were enriched among younger patients. Intriguingly, in our previous study on HNSCC, we detected the same two pathways enriched among older patients, which were the higher mutational rate samples due to the proportional relation between the HNSCC global mutational load and patient age [16]. Therefore, although the inverse tendency, Axon Guidance and ECM-Receptor Interaction pathways seem to show a relation with higher mutational rate squamous carcinomas. Several studies reported that Axon Guidance pathway is involved in lung cancer development and progression through interacting with cell survival, migration, and tumor angiogenic pathways [50–54]. Further studies are needed to determine whether disruptions in these pathways are a correlative phenotype to higher mutational rate squamous carcinomas or a causative factor.

In conclusion, multiple mutational processes appear to be simultaneously operative with various dynamic changes due to the endogenous and exogenous environments, life style habits and physiological ageing. Previous hypothesis of a mutator phenotype concealing the effect of age-related accumulation of mutations might have different causing factors in relation to ageing processes. We hypothesize that a higher sensitivity to smoking-related damages and the enrichment of defective DNA MMR SI6 may contribute to the higher mutational burden of younger patients. A higher overall level of methylation was as well detected in younger patients. While the defective DNA MMR SI26 showed increasing tendency along patient ageing. Therefore, the two distinct age-related defective DNA MMR signatures SI6 and SI26 might be crucial mutational patterns in LUSC tumorigenesis which may develop distinct phenotypes.

The evaluation of somatic genomic alterations along patients ageing might be relevant for a better comprehension of LUSC tumorigenesis and development of age-adjusted treatments.

## MATERIALS AND METHODS

### TCGA data sets

Multiplatform genomic data sets were generated by TCGA Research Network (http://cancergenome.nih.gov/). Cancer molecular profiling data were generated through informed consent as part of previously published studies [55] and analyzed in accordance with each original study’s data use guidelines and restrictions. The clinical data of the 504 LUSC normal paired exome sequences was derived via download from the publicly available GDC Data Portal (https://portal.gdc.cancer.gov/).

### Whole exome analysis

Somatic mutations were obtained from the open access MAFs available from the GDC Legacy Archive (https://portal.gdc.cancer.gov/legacy-archive). We considered three different exclusion criteria for mutation data entries. Samples belonging to the same patient share a very similar mutational profile. In the first exclusion criteria, we considered only once a mutation present in different samples belonging to the same patient. The mutations not included were equal to the 25.2% (282163 =>210948).

Some genes can share a similar sequence, such as paralogous genes. In presence of a mutation event on a sequence shared among different genes, it will not be possible to identify the mutated gene. With the second exclusion criterion, we decide to remove mutations that were associated to more than one gene. In this step we removed the 0.1% of mutations (210948 => 210700).

The challenges of repetitive sequence, which constitute 50–69 % of the human genome leads to false positive variant calls due to systematic sequencing errors and local alignment challenges [56]. Therefore, only somatic mutations with “ref context” containing less than 6 continuous single repetitions, less than 4 continuous duplets, less than 3 continuous triplets, less than 3 continuous quadruplets, less than 3 continuous quintuplets were kept. With the third exclusion criteria, the mutations were reduced from 210700 to 194170 (∼8.8%).

The patient TGCA-66-2755 was excluded from the following analysis due to the unusual number of mutations.

### SNP array-based copy number analysis

DNA from each tumor or germline-derived sample had been hybridized to Affymetrix SNP 6.0 arrays [57] and processed through GISTIC [58, 59] by the TCGA consortium.

High-level copy gain or copy loss events for individual genes were inferred using the publicly available Firehose’s (Gistic2.Level4) data (http://gdac.broadinstitute.org/runs/analyses__2016_01_28/data/LUSC/20160128/) (+2 values being indicative of gains greater than 1-2 copies, -2 values being indicative of near total copy loss). Global CNV load were calculated summing the absolute values from each patients.

### Array-based DNA methylation assay

DNA methylation profiles had been previously generated by TCGA using either the Infinium HM450 or HM27 assay probe. The level 3 beta value DNA methylation scores for individual genes were inferred using publicly available data generated by Illumina Human Methylation 450 platform downloaded from the GDC Legacy Archive (https://portal.gdc.cancer.gov/legacy-archive). Methylation values were mean centered and scaled to unit variance. After the transformation, the rate of methylation changes was calculated summing the values of each gene.

### Single nucleotide variants and COSMIC signatures

The signature profile was evaluated using the six subtype: C>A, C>G, C>T, T>A, T>C, and T>G (all substitutions were referred to by the pyrimidine of the mutated Watson-Crick base pair). Further, each of the substitutions was examined by incorporating information on the bases immediately 5’ and 3’ to each mutated base generating 96 possible single nucleotide variants (6 types of substitution x 4 types of 5’ base x 4 types of 3’ base). The profile of these 96 single nucleotide variants was considered as the results of the combination of the 30 different COSMIC signatures. The profile of each tumor sample can be represented by a unique contribution of each COSMIC signature as the following expression:

$$a_{1}\times \mathrm{SI}1+a_{2}\times \mathrm{SI}2+a_{3}\times \mathrm{SI}3+\&+a30\times \mathrm{SI}30$$ (1)

where *a*_*i*_ is the coefficient representing the contribution of the *i*_*th*_ COSMIC signature. The coefficients of each tumor samples were calculated minimizing the difference between the tumor profile and the expression *(1)*. This procedure was implemented using the function *optim* (method “L-BFGS-B” [60]) of the R software [61].

### Molecular pathway and biological process analysis

Pathway analyses were performed by ssGSEA using the GenePattern module ssGSEA Projection (v4) (genepattern.broadinstitute.org). ssGSEA enrichment scores were calculated from SNPs, CNV, and methylation LUSC data sets. The result is a single score per patient per gene set, transforming the original data sets into a more interpretable higher-level description. For the use of ssGSEA software, annotated gene sets reference were obtained from the C2 KEGG sub-collection of the Molecular Signature database (MSigDB) [62]. Silent mutations (point mutations that would not result in a change in the amino acid sequence) were not included in the analysis.

### Statistical analysis

The Spearman’s Rank Correlation Coefficient was used to identify correlation between patient age and genomic/epigenomic data (e.g., SNP, CNV, and methylation loads). For every Spearman’s test performed in this study, p-values were computed using algorithm AS 89 included in the R function *cor.test* where the permutation distribution was estimated by an Edgeworth approximation [63]. The coefficient interval of rho value was calculated by bootstraping (with 1000 replicates) using the function *spearman.ci* of the R package *RVAideMemoire*. Fisher’s exact test was used to examine the significance of the association between COSMIC signature related subgroups (i.e., low-SI6/high-SI26 and high-SI6/low-SI26) and clinical/demographic/molecular patient features, such as gender, tobacco smoking history indicator, and mutated / wild type genes. Fisher’s exact test was computed using the R function *fisher.test*. Wilcoxon Rank-Sum test was performed to compare continuous variables between two patient subgroups using the R function *wilcox.test*. A p-value <0.05 was considered to be significant. To account for multiple testing, a FDR of ≤20% was applied to reduce identification of false positives [64]. The FDR was calculated using the R function *p.adjust*. All calculations were made using R software [61].

## SUPPLEMENTARY MATERIALS TABLES




















