# Supplementary material for: Somatic genome alterations in relation to age in lung squamous cell carcinoma
Source: Oncotarget. 2018 Aug 14;9(63):32161–72. doi: 10.18632/oncotarget.25848 (PMC6114948; doi:10.18632/oncotarget.25848)
Supplement: Supplementary file 1 [file oncotarget-09-32161-s001.pdf]

Somatic genome alterations in relation to age in lung squamous cell carcinoma

SUPPLEMENTARY MATERIALS

Supplementary Table 1: SNPs dataset

Low impact mutation	*n*
RNA	10,755
Silent	42,414
Moderate impact mutation	*n*
Missense_Mutation	114,091
In_Frame_Ins	277
In_Frame_Del	1,454
High impact mutation	*n*
Splice_Site	6,807
Nonsense_Mutation	8,582
Translation_Strat_Site	684
Nonstop_Mutation	207
Frame_Shift_Del	3,771
Frame_Shift_Ins	2,108

Supplementary Table 2: SNPs, CNVs and methylation changes loads correlation with patient age.

See Supplementary File 1

Supplementary Table 3: Correlation of gene-specific alterations with patient age.

See Supplementary File 2

Supplementary Table 4: Correlation of somatic alterations in LUSC significantly mutated genes with patient age.

See Supplementary File 3

Supplementary Table 5: Mutator phenotype analysis.

See Supplementary File 4

Supplementary Table 6: SNPs trinucleotide correlations with patient age.

See Supplementary File 5

Supplementary Table 7: Age-related COSMIC signatures.

See Supplementary File 6

Supplementary Table 8: Characterization of low-SI6/high-SI26 and high-SI6/low-SI26 subgroups.

See Supplementary File 7

Supplementary Table 9: Gene Set Enrichment Analysis – low-SI6/high-SI26 and high-SI6/low-SI26 subgroups.

See Supplementary File 8

Supplementary Table 10: Gene Set Enrichment Analysis – global cohort.

See Supplementary File 9
